# Adenovirus and Immunotherapy: Advancing Cancer Treatment by Combination

**DOI:** 10.3390/cancers12051295

**Published:** 2020-05-21

**Authors:** Mizuho Sato-Dahlman, Christopher J. LaRocca, Chikako Yanagiba, Masato Yamamoto

**Affiliations:** 1 Division of Basic and Translational Research, Department of Surgery, University of Minnesota, MMC 195, 420 Delaware St SE, Minneapolis, MN 55455, USA; satom@umn.edu (M.S.-D.); clarocca@umn.edu (C.J.L.); yanag010@umn.edu (C.Y.); 2 Masonic Cancer Center, University of Minnesota, Minneapolis, MN 55455, USA; 3 Division of Surgical Oncology, Department of Surgery, University of Minnesota, Minneapolis, MN 55455, USA; 4 Institute of Molecular Virology, University of Minnesota, Minneapolis, MN 55455, USA

**Keywords:** adenovirus vector, oncolytic adenovirus, immunotherapy, checkpoint inhibitor, CAR-T cell therapy

## Abstract

Gene therapy with viral vectors has significantly advanced in the past few decades, with adenovirus being one of the most commonly employed vectors for cancer gene therapy. Adenovirus vectors can be divided into 2 groups: (1) replication-deficient viruses; and (2) replication-competent, oncolytic (OVs) viruses. Replication-deficient adenoviruses have been explored as vaccine carriers and gene therapy vectors. Oncolytic adenoviruses are designed to selectively target, replicate, and directly destroy cancer cells. Additionally, virus-mediated cell lysis releases tumor antigens and induces local inflammation (e.g., immunogenic cell death), which contributes significantly to the reversal of local immune suppression and development of antitumor immune responses (“cold” tumor into “hot” tumor). There is a growing body of evidence suggesting that the host immune response may provide a critical boost for the efficacy of oncolytic virotherapy. Additionally, genetic engineering of oncolytic viruses allows local expression of immune therapeutics, thereby reducing related toxicities. Therefore, the combination of oncolytic virus and immunotherapy is an attractive therapeutic strategy for cancer treatment. In this review, we focus on adenovirus-based vectors and discuss recent progress in combination therapy of adenoviruses with immunotherapy in preclinical and clinical studies.

## 1. Introduction

Cancer immunotherapy is a promising therapeutic modality for cancer which functions through stimulating the immune system to specifically target and attack cancer cells. There are many forms of immunotherapies including immunostimulatory cytokines, checkpoint inhibitors, antibodies, chimeric antigen receptor (CAR) T cells, and viro-immunotherapy [1]. While many of these therapies have been FDA-approved and showed increased patient survival rates compared to standard chemotherapies (i.e., CTLA-4 mAb), immunotherapy still has limitations. For example, dose limiting toxicities and unwanted responses from the immune system result in resistance to treatment or off-target attack of healthy cells [2,3]. In the case of immune checkpoint inhibitors, tumor associated macrophages change the immune composition of the tumor microenvironment, decreasing the effect of anticancer immune cell recruitment, and halting therapeutic effects [4]. Thus, for a variety of cancers, including many solid tumors, a novel approach for immunotherapeutic agents are necessary to increase the efficacy of penetrating physical barriers in the tumor microenvironment such as cancer associate fibroblast (CAF), and mitigating adverse effects associated with current immunotherapeutic agents.

Virotherapy is a novel and versatile form of cancer therapeutics and can be utilized as a vaccine to stimulate the immune system, as well as for direct oncolysis induced by viral replication. The natural antiviral immune response can be utilized to reprogram the tumor microenvironment from “cold” to “hot” by inducing a T cell mediated response against cancer cells [5]. This will increase immune cell accessibility within the tumor microenvironment, allowing for better delivery/infiltration of cytokines and T cells. Additionally, selective elimination of cancer cells by replicative oncolytic viruses can be achieved through genetic modifications that enhance virus-tumor tropism and intratumoral viral amplification [6]. 

A prime example of oncolytic viral therapy is T-VEC (talimogene laherparepvec), the only FDA-approved oncolytic virus. This modified herpes simplex 1 virus, is primarily used for treatment of metastatic melanoma [7]. T-VEC is designed to selectively replicate and lyse melanoma cells, while also producing granulocyte macrophage colony-stimulating factor (GM-CSF) to enhance systemic antitumor immune responses [7]. GM-CSF promotes maturation of dendritic cells (DCs) and activation of T cells via antigens [8,9]. One clinical trial tested the combination of T-VEC with anti-PD-1 antibody, pembrolizumab, in patients with advanced melanoma. At an overall response rate of 62%, patients showed increased levels of cytotoxic CD8+ T cells compared to patients who only had PD-1 antibody treatment [10]. 

One of the most well-studied and promising viral vectors is adenoviruses (Ads). It can be widely used in two forms: replicative oncolytic adenoviruses and replicative deficient adenoviruses for vaccines and gene therapy [11,12]. There are many characteristics of the adenovirus which make it favorable viral vector. Firstly, the genome of the virus is well-understood, genetically stable, and has a large cloning capacity. Ads have a high in vivo transduction efficiency in humans, and the side effects can be very mild compared to chemotherapies, and even some forms of immunotherapy due to not integrating into the hosts’ genome [13]. While the natural receptor of adenovirus, coxsackievirus and adenovirus receptor, is not expressed in many cancers, the fiber/knob domain of the virus capsid can be genetically manipulated to redirect adenovirus binding to different cell surface receptors [12]. Furthermore, Ad is a nonenveloped virus with a replication cycle culminating in lysis of host cells. This lytic replication cycle is favorable for oncolysis [14] when compared to enveloped viruses which complete replication by budding from intact, live, host cells. In this sense, Ad-based therapy is a one of the most promising anticancer agents and has been employed for its antitumor potency via intratumoral amplification and strong oncolytic effect. Additionally, Ad can infect dendritic cells and upregulate immunostimulatory signals, which can effectively present antigens to the immune system [15]. Indeed, a number of studies are currently underway exploring the prospect of Ad as a vaccine against cancer or infectious diseases such as HIV. Thus, Ad is an ideal vector for use in cancer treatment through different forms as a replicating oncolytic vector, or as a vaccine vector. 

While preliminary trials of Ad-based therapy are promising, further improvements can increase its efficacy as a cancer treatment. Coadministration with immunotherapy will recruit the host patient’s antitumor immune response to attack cancer cells. In this review, we will discuss potential strategies and therapeutic effects of Ad-based therapies by combination of immunotherapeutic reagents in preclinical and clinical studies. 

## 2. Adenovirus Vector as a Therapeutic Cancer Vaccine

**Figure 1:**
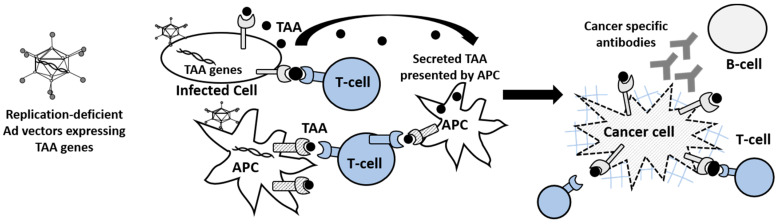
Cancer vaccine based on replication-deficient adenoviral vectors. The injection of tumor-associated antigens (TAAs) encoded nonreplicative Ads into nontumor tissue or antigen-presenting cells (APCs). TAAs can be either expressed on MHC (Major Histocompatibility Complex) class I at the surface of the infected cells, or secreted and presented by APCs. If Ad infects APCs, encoded TAAs express on MHC class II at the surface of APCs.

The concept of a cancer vaccine is to train the immune system to mount an attack against cancer cells in the body. Cancer vaccines include synthetic peptides, recombinant proteins, nucleic acids, autologous cells, and bacterial or recombinant viral vectors. Among the viral vectors, replication-deficient adenovirus vectors have been employed extensively as vaccines because they induce strong humoral and T cell responses to transgenes expressed by the vector [11] (Figure 1). Currently, some of the Ad vector vaccines are being investigated in human clinical trials for cancer.

### 2.1. ETBX-011 (Ad5-CEA) 

Carcinoembryonic antigen (CEA) is a glycoprotein expressed in a variety of cancers. ETBX-011 is an engineered adenoviral vaccine encoding a modified CEA containing the highly immunogenic epitope CAP1-6D, induced CEA-specific cell-mediated immune responses with antitumor activity [16]. ETBX-011 has deletion of the E1- (E1A and E1B), E2B- (the viral polymerase), and E3-genes [17,18], with expression of CEA driven by the cytomegalovirus (CMV) promoter. Potent expression of CEA induced CEA-specific cell-mediated immune responses with antitumor activity. Multiple phase I/II trials with this vector are currently underway (NCT03563157, NCT03387098) [18,19]. ETBX-011 was well tolerated in metastatic colorectal cancer patients, and CEA-directed T cell responses were induced in a dose-dependent manner. This study exhibited evidence of a potential survival benefit: 25 patients treated at least twice with ETBX-011 exhibited a 12-month overall survival probability of 48% and a mean overall survival of 11 months [19,20].

To overcome the phenomenon of tumor heterogeneity, including diversity of tumor-associated antigen (TAA) expression, a combination of ETBX-011 with two different human TAA-expressing Ad vector vaccines have demonstrated antitumor cytotoxic T cell (CTL) responses in preclinical animal models of cancer [21]. The first, ETBX-061, is an Ad5-based adenovirus vector vaccine with the same backbone as the ETBX-011, but expressing a modified human mucin 1 (MUC1) gene. The modified MUC1 gene contains agonist epitopes designed to increase CTL antitumor immune responses [21]. The other, ETBX-051 or Ad5-brachyury, encodes the entire brachyury gene with a deletion of 25 amino acids involved in DNA binding, and modified to express an enhancer T cell epitope. The recently reported phase I clinical trial with triple combination of Ad vaccine (ETBX-011, ETBX-061, and ETBX-051; Tri-Ad) demonstrated that the Tri-Ad vaccine regimen is safe and well tolerated (NCT03384316) [22].

### 2.2. Ad5-PSA

Ad5-PSA is replication-deficient adenovirus type 5 carrying the human prostate-specific antigen (PSA) gene. In a preclinical mouse model of prostate cancer, this Ad vaccine induced strong anti-PSA T cell responses and caused the destruction of PSA-secreting tumor cells [23]. In the subsequent phase I clinical trial, the Ad5-PSA vaccine was proven safe with no serious vaccine-related adverse events. The majority of vaccinated patients produced anti-PSA T cell responses, and over half survived longer than predicted by nomogram [24]. In an ongoing phase II clinical trial, patients with recurrent or hormone refractory prostate cancer are being treated with Ad5-PSA (NCT00583024). To date, 100% of patients with recurrent disease and 67% of patients with hormone refractory disease demonstrated anti-PSA T cell responses. [25,26] A newly recruiting phase I clinical trial will examine how patients with metastatic castration resistant prostate cancer respond to adenoviral PSA, MUC1 (ETBX-061), and ETBX-051 (brachyury) vaccines (NCT03481816).

### 2.3. Ad-E6E7 and Ad-MAGEA3

For the treatment of HPV-positive cancers, a current phase I clinical trial is using the replication-deficient adenovirus-based vaccine, Ad-E6E7 [27], in combination with the oncolytic maraba virus strain MG1 vaccine, MG1-E6E7, and sequential treatment with atezolizumab (anti-PD-L1 antibody). Both Ad-E6E7 and MG1-E6E7 express human papillomavirus (HPV) genes E6 and E7, altered to remove regions required for oncogenic transformation [28]. In a preclinical study, immune priming with Ad-E6E7, subsequently boosted with MG1-E6E7, mounted tumor-specific responses of remarkable magnitude, which significantly prolonged survival in various mouse cancer models [29]. Ad-E6E7 has been investigated in phase I clinical trial with atezolizumab (anti-PD-L1 antibody) in HPV associated cancer patients (NCT03618953).

Using the same strategy, there are two phase I/II clinical trials evaluating the Ad:MG1 prime-boost combination as an oncolytic cancer vaccine platform. The first utilizes the oncolytic maraba MG1-MAGEA3 (melanoma-associated antigen A3) vaccine, either alone or following adenovirus-MAGEA3, for treatment of patients with advanced MAGE-A3-positive solid tumors (NCT02285816). The second is evaluating the oncolytic MG1-MAGEA3 with Ad-MAGEA3 vaccine in combination with pembrolizumab (anti-PD-1 antibody) for patients with previously treated metastatic non-small cell lung cancer (NCT02879760). 

In summary, viral vector-based cancer vaccines, including adenovirus-based vaccines, provide promising therapeutic strategies to activate antitumor immune responses. Although numerous cancer vaccine strategies have been studied, therapeutic vaccines still show very few positive outcomes in the establishment of clinical responses in advanced cancer patients when used alone. The key barriers to their success are tumor heterogeneity and low antigenicity of targeted antigens. To overcome these issues, a number of combination approaches, such as administration of multiple types of viral vaccinations or using cancer vaccines in combination with immune checkpoint inhibitors, have been tested in both preclinical and clinical trials in the cancer vaccine field [30]. Further results from these ongoing clinical trials are awaited.

## 3. Immunostimulatory Molecules-Expressing Adenovirus Vectors

**Figure 2:**
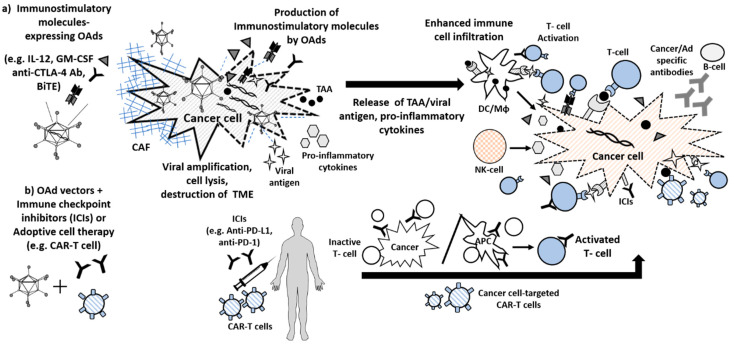
Oncolytic adenovirus-based cancer immunotherapies. (**a**) Immunostimulatory molecules-expressing oncolytic Ad vectors. Local immunostimulatory molecules expression by oncolytic adenoviruses (OAds) (such as interleukin 12 (IL-12), granulocyte macrophage colony-stimulating factor (GM-CSF)) enhances APCs migration and maturation, leading to T cell activation. Anti-CTLA-4 antibodies blocks inhibitory signals and restore T cell activation. BiTE molecule can bridge T cells and tumor cells, thereby promoting T cell activation to kill cancer cells. Furthermore, OAd can induce strong antitumor immunity through direct oncolysis and tumor antigen release. In addition, viral oncolysis causes destruction of physical/anatomical barriers, such as cancer associate fibroblast (CAF), leading to change the tumor microenvironment (TME) from “cold” tumors to “hot”. (**b**) Combination with OAds and immune checkpoint inhibitors (ICIs) or adaptive cell therapy. Blocking the binding of immune checkpoint signal with an immune checkpoint inhibitor (such as anti-PD-L1, anti-PD-1 and anti-CTLA-4) allows the T cells to be active and to kill cancer cells. When cancer-targeted chimeric antigen receptor (CAR) T cells are infused into the body, the CAR-T cells can bind to an antigen on the target cancer cells and kill them.

Several viruses have been engineered to express different immune modulators, or cytokines. One of the most frequently used cytokines for arming oncolytic viruses is GM-CSF. Local GM-CSF expression by oncolytic virus enhances DC migration and maturation, leading to T cell activation [31] (Figure 2a). GM-CSF armed oncolytic herpes simplex virus (T-VEC; talimogene laherparepvec) has been approved by the FDA for treatment of metastatic melanoma patients [32,33]. Other oncolytic viruses have also been successfully armed with GM-CSF, including various adenoviruses (e.g., ONCOS-102, described in more detail in the next chapter).

### 3.1. IL-12-armed Ads

Another cytokine that has been used for arming Oncolytic virus is interleukin 12 (IL-12). IL-12 induces a potent antitumor effect by promoting natural killer (NK) cell and cytotoxic T cell activities [34,35]. Ads armed with IL-12 have shown enhanced antitumor effect and immune stimulation in preclinical and clinical studies (murine [36] and Syrian hamster models [37], and in clinical trials [38]). Wang et al. was able to decrease the toxicity of systemic accumulation of IL-12 by cleaving the N-terminal signal peptide of IL-12 so that IL-12 was not secreted but was delivered directly to the tumor by oncolytic adenovirus (Ad-TD-nsIL-12). This adenovirus mediated delivery of nonsecreting IL-12 successfully treated pancreatic cancer in Syrian hamster models, without toxicity. Additionally, the virus provided long-term immunity as evidenced by resistance to tumor rechallenge [39].

Oncolytic or replication-deficient adenovirus-based vectors expressing IL-12 are currently in clinical trials. The oncolytic adenovirus Ad5-yCD/mutTKSR39rep-hIL12, encodes the human IL-12 gene with two suicide fusion genes: a yeast cytosine deaminase (yCD), and a mutant form of herpes simplex virus type 1 thymidine kinase (HSV-1 TKSR39) with potential immunomodulating and antineoplastic activities. Ad5-yCD/mutTKSR39rep-hIL12 is in phase I clinical trials for locally recurrent prostate cancer and metastatic pancreatic cancer (NCT02555397, NCT03281382).

Ad-RTS-hIL-12 is a replication-deficient adenoviral vector encoding human IL-12 p70 transgene under the control of the RheoSwitch Therapeutic System (RTS). In this system, transcription of the IL-12 transgene occurs only in the presence of the activator ligand, veledimex (Ziopharm Oncology) [40,41]. Several phase 1 trials have examined Ad-RTS-hIL-12 in brain tumors, glioblastoma, or malignant glioma (NCT03330197, NCT02026271). A recent trial found regulated expression of recombinant IL-12, following intratumoral injection of Ad-RTS-hIL-12 in combination with oral veledimex, to be safe in 31 patients with recurrent glioblastoma [42]. In addition to IL-12, several other interleukins, including IL-2, IL-24 and IL-13, have been used to arm oncolytic adenovirus and have shown promising immune-activating properties in multiple preclinical cancer models [43,44,45]. 

### 3.2. LOAd703

Another approach, using oncolytic adenovirus LOAd703 armed with immunostimulatory genes, has been reported. LOAd703 is armed with 4-1BBL and trimerized CD40L, which activate the CD40 and 4-1BB pathways, respectively [46]. LOAd703 viruses were able to replicate and kill pancreatic cancer cells via oncolysis in both in vitro and in vivo assays [46]. A clinical trial is ongoing to investigate the safety and efficacy of repeated LOAd703 intratumoral injections in combination with standard of care in pancreatic and ovarian cancer patients (NCT02705196, NCT03225989).

### 3.3. DNX-2440 (OAd-OX40L) 

DNX-2440 is an armed oncolytic adenovirus expressing the immune costimulator OX40 ligand (OX40L), which is combined with the virus backbone of DNX-2401(d24-RGD). Phase I clinical trial of DNX2440 is currently ongoing for patients with glioblastoma (NCT03714334). 

### 3.4. Ad5/3-Δ24aCTLA4

Ad5/3-∆24-based oncolytic adenovirus (OAd) coding for anti-CTLA4 antibody has been tested in several cancer cell lines [47]. Anti-CTLA4 antibodies are designed to block the interaction of CTLA-4 with its ligands, and prevent inhibition of T cell function. Promising clinical results have been achieved with anti-CTLA4 antibodies ipilimumab and tremelimumab [48,49,50]. Ipilimumab was approved by the U.S. FDA in 2010 for the treatment of advanced melanoma [51]. However, systemic administration of these agents also has the potential for severe immune-related adverse events [49,52,53]. Local expression of anti-CTLA4 antibody induced by administration of armed oncolytic adenovirus resulted in significantly higher concentrations of the antibody in the tumor, while plasma levels remained at safe concentrations, as indicated in mouse models [47]. T cells of patients, but not those of healthy donors, were activated by an anti-CTLA4 antibody produced by Ad5/3-∆24-CTLA4. Furthermore, a direct anti-CTLA4-mediated proapoptotic effect was observed in vitro and in vivo [47]. 

### 3.5. OAd-IFN-α

Interferon-α (IFN-α) based therapy regimens are being pursued as promising tools in treating pancreatic cancer. IFN-α has a strong antitumor effect, and has the ability to sensitize cancers to the tumoricidal effects of chemo-radio therapy [54,55,56,57,58]. Several clinical trials have described adjuvant IFN-α therapy in the treatment of pancreatic cancer. Some studies reported a remarkable increase in 2- and 5-year survival rates [54,59,60]. Despite encouraging survival statistics and immunological data, limitations to IFN-based therapies include dose-limiting systemic toxicities and low intratumoral concentration of IFN due to its short half-life in the bloodstream [61,62].

Our group has previously reported the use of oncolytic adenovirus expressing human IFN-α as a promising platform for selective, long-term expression of IFN in human pancreatic cancer tissues [63,64]. This conditionally replicative adenovirus (Ad5/Ad3-Cox2-ΔE3-ADP-IFN) was designed to selectively replicate within cancer cells expressing cyclooxygenase 2 (Cox2). To improve the infectivity and oncolysis of conventional oncolytic adenoviruses (OAds), the virus was genetically modified to include an Ad5/Ad3 chimeric fiber and overexpress the adenovirus death protein (ADP). In our later studies, we tested another OAd (OAd-hamIFN) in combination with chemo and radiation therapies in an immunocompetent Syrian hamster model of pancreatic ductal adenocarcinoma (PDAC) [65,66]. As a result, OAd with IFN-α showed efficient viral replication in tumor, significant inhibition of tumor growth, and enhanced survival [65,66].

### 3.6. OAd with BiTE

It is worth highlighting that arming oncolytic viruses with bispecific T cell engagers (BiTEs) realizes a combined anticancer therapy. BiTE antibody constructs comprise tandemly arranged single-chain variable fragments (scFvs); one binds to CD3ε subunit on the T cell receptor, while the other binds to a chosen target antigen. The first BiTE-armed oncolytic virus that has been tested in preclinical models is an oncolytic vaccinia virus armed with a BiTE molecule targeting the tumor cell surface antigen EphA2 (EphA2-TEA-VV) [67]. EphA2-TEA-VVs displays significantly enhanced antitumor activity by inducing bystander killing of noninfected tumor cells, compared to unmodified oncolytic VVs in vitro assay. Additionally, EphA2-TEA-VV plus human peripheral blood mononuclear cells (PBMCs) displayed potent antitumor activity in a lung cancer xenograft model [67].

Likewise, an oncolytic adenovirus expressing an EGFR-targeting BiTE was engineered by the Alemany group [68]. The anti-EGFR BITE-armed OAd showed improved T cell-mediated killing of cancer cells both in vitro and in vivo [68]. The group also reported that combination of anti-EGFR BiTE-armed OAd in combination with adoptive CAR-T cell therapy resulted in improved antitumor efficacy and prolonged survival in mouse models as a result of intratumoral T cell activation by BiTE [69]. 

The Fisher group has also generated an armed oncolytic adenovirus to express a BiTE molecule which binds to epithelial cell adhesion molecule (EpCAM), a surface protein overexpressed on targeted cancer cells (EnAd-SA-EpCAM) [70]. This virus has shown to effectively activate endogenous T cells within the immune-suppressive microenvironment and exhibited killing of endogenous tumor cells without the addition of exogenous T cells [70]. Recently, this group reported development of a stroma-targeted BiTE-expressing adenovirus (EnAd-FAP-BiTE). Fibroblast activation protein (FAP) is highly overexpressed in cancer-associated fibroblasts (CAFs). CAFs are the main cellular component of stroma in a solid tumor microenvironment (TME). Their FAP-BiTE-encoding virus induced the activation of tumor-infiltrating PD-1^+^ T cells which target and kill CAFs [71]. Similar results were reported by the Alemany group using the FAP-BiTE-armed oncolytic adenovirus (ICO15K-FBiTE). ICO15K-FBiTE is shown to enhance viral spread and overall antitumor efficacy without increasing the toxicity in mouse model [72].

A combination of therapies taken together using adenovirus and immunostimulatory molecules is an appealing strategy. Expression of immunologic effector molecules, such as GM-CSF, IL-12, CD40L, and IFN-α, allows oncolytic adenoviruses to become chaperones of these key proinflammatory cytokines and chemokines, which accumulate in a selectively targeted area within the tumor [73]. While several promising immunostimulatory-encoding Ads have been tested in preclinical models, the development of a combinatory approach is a complex undertaking. While combination of treatment approaches can yield results superior to any individual approach, proper evaluation of combination effects is not straightforward, and determining appropriate timing, dosing and sequence schedules of each individual agent is crucial to the success of the whole. Unfortunately, there are not currently adequate animal models which permit both human adenovirus replication and detailed immunologic analyses, allowing prediction of immunological effects in patients. To date, most in vivo experiments of OAds have been performed in immuno-deficient mice bearing human tumor xenografts. Murine cancer models cannot be used because mouse cells do not permit replication of human Ads. However, the necessity of immuno-deficient animals in xenograft models will not reflect a host’s immune response against virus therapy, and will not accurately predict the virus-induced antitumor immune response in patients. Therefore, investigations into alternative animal models and well-controlled clinical trials have been pursued concomitantly in order to fully assess patient safety of OAds carrying immunostimulatory molecules. Establishment of combination therapies using adenovirus and immunostimulatory molecules has the potential to significantly impact clinical efficacy in a variety of cancers.

## 4. Oncolytic Adenovirus and Immune Checkpoint Inhibitors

Clinical use of immunotherapies as anticancer agents, such as immune checkpoint inhibitors and CAR-T cells, has grown rapidly in the last decade. Despite this progress, the low response rates observed in patients receiving immune checkpoint inhibitors. Studies confirmed that the response to immune checkpoint inhibitor therapy is related to tumor-infiltrating lymphocytes (TILs) and other immune cells in the tumor microenvironment (TME) [74]. Therefore, the changes to the local TME (from “cold” tumors into “hot”) following oncolytic adenovirus delivery creates a situation that can be exploited with novel combination strategy of oncolytic adenovirus and immune checkpoint inhibitors (Figure 2b).

### 4.1. DNX-2401 (Tasadenoturev)

DNX-2401 is a replication-competent oncolytic adenovirus that contains a 24 base pair deletion (Δ24) in the *E1A* gene which is designed to confer selective replication in cancer cells lacking the normal retinoblastoma (Rb) protein signaling pathway [75]. In addition, the infectivity of this virus is augmented by incorporating an RGD-4C motif into the adenoviral fiber HI-loop which allows for improved binding to the surface of cancer cells (as the native adenovirus receptor (coxsackie adenovirus receptor—CAR) is poorly expressed on many human cancers) [76]. This virus was tested in a phase 1 clinical trial in patients with recurrent, malignant gliomas, as these tumors harbor alterations in the Rb protein signaling pathways [77]. There were no dose-limiting toxicities, adenoviral shedding was minimal (<3% of post-treatment blood, urine, and sputum samples contained viral DNA), and 55% of resected tumors (performed on day 14 after injection) demonstrated active viral replication when they were analyzed for viral E1A or hexon proteins [77]. DNX-2401 has also been tested in a multicenter, phase II, dose-escalation clinical trial (CAPTIVE Study, Keynote-192, NCT02798406) in combination with intravenous pembrolizumab (PD-1 immune checkpoint inhibitor) in 48 patients with recurrent glioma [78]. Patients received a single, intratumoral dose of the virus (mostly commonly 5 × 10^10^ vp) and went on to receive the first dose of intravenous pembrolizumab 7 days after viral injection. At an interim analysis, the median overall survival was 12 months, and 47% of patients had stable or improved disease burden [78].

### 4.2. ONCOS-102 (Ad 5/3 Δ24 GM CSF)

ONCOS-102 is an oncolytic adenovirus that incorporates a GM-CSF transgene to augment the immune response, the chimeric Ad5/3 fiber knob modification to enhance viral infectivity, and a 24 base pair deletion in the E1A region of the genome (Δ24) resulting in selective viral replication in Rb-pathway deficient cells [79]. After extensive preclinical testing, this virus was utilized in a phase I clinical trial in 12 patients with advanced solid tumors including colon, lung, and ovarian cancers [80]. The results of this trial demonstrated no observed dose-limiting toxicities, and a strong immune cell infiltrate into tumors as evidenced by a 4.0- and 2.5-fold post-treatment increase in CD8+ and CD4+ T cells, respectively, as well as the presence of tumor-specific CD8+ T cells [80]. Interestingly, there was upregulated PD-L1 expression on the tumors of pleural mesothelioma patients following viral delivery, and this observation suggested that ONCOS-102 could prime the local immune microenvironment for subsequent immune checkpoint blockade [80]. To this end, an ongoing clinical trial is investigating the combination of ONCOS-102 with pembrolizumab for those patients with locally advanced or unresectable melanoma who progressed on PD-1 blockade (NCT03003676). Patients received three intratumoral injections (3 × 10^11^ vp; Day 1,4,8) followed by pembrolizumab (day 22 and every 3 weeks thereafter until week 27). Interim results of the first portion of the trial demonstrated that none of the nine participating patients had dose limiting toxicities, and 33% of these individuals demonstrated disease stability or regression on cross-sectional imaging [81]. In addition, all patients demonstrated increases in circulating proinflammatory cytokines, CD8+ T cells, and PD-1+ CD8+ T cells [81]. Of the 7 patients who had paired tumor biopsies, all had intra-lesional CD8+ T cells, and 6/7 patients had PD-1+ CD8+ T cells. Furthermore, 4 patients had either *de novo* development or increased levels of tumor specific T cells (MAGE-A1, NY-ESO-1) during the trial [81].

### 4.3. TILT-123 (Ad5/3-E2F-d24-hTNF-α-Internal Ribosome Entry Site [IRES]-hIL-2)

TILT-123 is an oncolytic adenovirus that incorporates transgenes for human tumor necrosis factor alpha (TNF-α) and interleukin-2 (IL-2) in addition to Ad5/Ad3 chimeric fiber designed to optimize transduction efficiency, and a 24 base pair deletion in *E1A* to confer selective replication in cells lacking a normal retinoblastoma/p16 pathway [82]. The Hemminki group chose these two cytokines in part due their previous work demonstrating how IL-2 and TNF-α are promising T cell stimulating factors in relation to adoptive cell therapy [83,84]. In addition, nonreplicating vectors expressing IL-2 (Ad5-CMV-mIL2) and TNF-α (Ad5-CMV-mTNF- α) have been tested in conjunction with programmed cell-death protein 1 (PD-1) blocking antibodies in a mouse model [85]. Notably, they demonstrated complete regression of murine melanoma (B16.OVA) tumors, and Improved survival of those animals treated first with intratumoral viral injections, followed by systemically-delivered anti-PD-1 antibodies (prime-boost approach) [85]. They also reported viral infection shifted the cytokine profile of the tumor microenvironment towards T-helper type 1 (Th1) and suggest that this could result in a favorable inflammatory status with improved antitumor effects [85]. Given these encouraging preclinical findings, the TILT-123 virus will be employed in multiple clinical trials. One of these will employ it in conjunction with tumor infiltrating lymphocyte (TIL) therapy (NCT04217473) for patients with advanced melanoma and an additional trial is expected where the TILT-123 virus will be combined with antiprogrammed death ligand-1 (PD-L1) and anti-PD-1 antibodies for patients with advanced melanoma and other solid tumors.

The combination of oncolytic adenoviruses and immune checkpoint inhibitors have shown great promise in recent years as evidenced by the response seen in completed and ongoing clinical trials. These therapies will continue to evolve together, and approaches will need to be refined to optimize outcomes for patients with advanced cancers.

## 5. Oncolytic Adenovirus and Immunotherapy with Chimeric Antigen Receptor (CAR) T Cells 

Adoptive cell therapy (ACT) is one type of immunotherapy that includes the use of tumor-infiltrating lymphocytes (TIL), T cell receptor modified (TCR) T cells, and chimeric antigen receptor (CAR) T cells [86]. TCR-T cells are designed to encode receptors for select cancer-specific antigens, and function through an MHC-dependent mechanism, which is one limitation to their use [87]. CAR-T cell therapy functions through an MHC (Major Histocompatibility Complex)-independent mechanism and has been remarkably effective in the treatment of hematologic malignancies, including chronic lymphocytic leukemia and non-Hodgkin’s lymphoma [88,89]. However, it has had limited success in solid tumors due to many factors, including poor tumor infiltration of CAR-T cells and an immunosuppressive tumor microenvironment (TME) [90]. One approach to overcoming these obstacles of CAR-T therapy in solid tumors is to utilize CAR-T cells in conjunction with oncolytic viruses as part of a combination regimen (Figure 2b). Oncolytic viruses have the ability to infect targeted cells of interest, selectively replicate within them, and deliver transgenes. Furthermore, oncolytic viruses modulate the TME to increase immune cell infiltrates, and have been extensively tested in preclinical and clinical trial settings with an excellent safety profile [91]. In addition, oncolytic virus treatment may be able to mitigate the cancer-protective action of tumor stroma, which often serves as physical and/or anatomical barriers preventing therapeutic agents’ access to cancer cells [92].

The immunosuppressive nature of the pancreatic cancer TME limits the effectiveness of T cell infiltration and induces T cell hypofunction, leading Watanabe et al. to describe the combination of oncolytic adenoviruses (expressing TNF-α and/or IL-2) and mesothelin-redirected CAR-T cells (meso-CAR-T) in an attempt to overcome these limitations [93]. In an NOD/SCID/Il2rg null (NSG) xenograft mouse model using AsPC-1 cells, they demonstrated that tumors treated with the combination of the virus expressing TNF-α and IL-2 (Ad5/3-E2F-d24-TNF-α-IRES-IL-2 [OAd-TNFα-IL2]) along with meso-CAR-T cells were infiltrated with significantly more CD4+ and CD8+ T cells compared to monotherapy with meso-CAR-T cells, or a combination with meso-CAR-T cells and the parent adenovirus lacking cytokine expression (OAd) [93]. They also demonstrated that meso-CAR-T cells in combination with OAd-TNFα-IL2 resulted in significantly higher accumulation of CAR-T cells at the tumor site (when compared to meso-CAR-T monotherapy or meso-CAR-T cells and OAd in combination) as seen on bioluminescent imaging (evident as early as two days following injection and persisting for up to 50 days), as well as improved tumor regression [93]. The authors also employed a syngeneic, immunocompetent mouse model to more closely replicate the TME of human pancreatic cancer. They found that the mouse tumors were resistant to even multiple doses of antimouse mesothelin CAR-T cells (meso-CAR-T), but combination therapy of meso-CAR-T with Ad-mTNFα-mIL2 yielded robust and statistically significant tumor suppression, thereby highlighting the importance of adenoviral mediated cytokine delivery for CAR-T therapy [93].

The Suzuki group has employed another strategy to improve the efficacy of CAR-T cell therapy utilizing a combinatorial adenovirus vector (oncolytic adenovirus (Ad5Δ24) and helper-dependent adenovirus expressing a mini anti-PD-L1 antibody (HDAdPD-L1) collectively termed CAd-VEC*PDL1*) in conjunction with human epidermal growth factor receptor 2 (HER2)-specific CAR-T cells [94]. Using this combination in an NSG mouse model of prostate cancer xenograft demonstrated that while in the presence of HER2-CAR-T cells, the CAd-VEC*PDL1* virus’s expression of anti-PD-L1 antibody at the tumor site was significantly more effective at reducing tumor size. In addition, the mice had 2-fold higher median survival (110 days) when compared to the systemically administered anti-PD-L1 antibody monotherapy [94]. While other research groups have demonstrated the utility of cytokine-expressing adenovirus vectors to enhance CAR-T cell therapy, the Suzuki group further modified the CAd-VEC*PDL1* vector by incorporating IL-12 (CAdVEC*IL12*_*PDL1*) and tested it in a head and neck squamous cell carcinoma (HNSCC) model, as these tumors are often more resistant to CAR-T therapy [95,96]. In a xenograft model of NSG mice using SCC-47 or FaDU cell lines, the combination of HER2-CAR-T cells and CAdVEC*IL12*_*PDL1* virus significantly prolonged survival of treated animals to more than 100 days, compared to 21–24 days in the control groups, and HER2-CAR-T cells were detected in the tumors of surviving mice over 100 days after initial therapy [96]. The research group also used an orthotopic HNSCC model, establishing both primary tumors and lymphatic metastases, to test the aforementioned combination therapy. Mice that received both HER2-CAR-T cells and CAdVEC*IL12*_*PDL1* had improved tumor growth control at both primary and metastatic sites, maintained body weight, and had prolonged survival when compared to untreated and monotherapy groups [96]. Interestingly, HER2-CAR-T cells were detected at the primary and metastatic tumor sites up to 120 days following intravenous delivery and maintained their HER2-CAR expression, while adenovirus DNA was only detectable at the primary lesion. These results suggest that the HER2-CAR-T cells play a significant role in controlling metastatic disease burden, but require adenovirus-mediated transgene expression of cytokines for optimum efficacy [96].

CAR-T cells as a monotherapy have not demonstrated much success in solid tumors, but combination therapy with oncolytic viruses provides one potential strategy for improvement. These viruses can deliver cytokines or other immunomodulatory molecules to enhance the effect of CAR-T cells, and early results are encouraging. Defining the optimum choice for oncolytic virus-delivered molecules to be combined with each unique CAR-T cell therapy will be a key step towards targeting and treatment of specific tumor types.

## 6. Conclusions

Backed by growing evidence from preclinical and clinical studies, it is now well accepted that combining oncolytic virus with immunotherapeutic agents significantly amplifies the therapeutic effect of both agents. There are multiple ongoing clinical trials that are currently using adenovirus-based vectors for cancer immunotherapy (Table 1).

While the prospect of using oncolytic adenoviruses (OAds) for cancer immunotherapy is appealing, it also has its limitations. The majority of oncolytic viruses, including OAds, provides moderate antitumor effects as a monotherapy. This is most likely due to the tumor microenvironment, antiviral response as well as neutralizing antibodies [97]. In particular, the high prevalence of pre-existing neutralizing antibody against adenovirus type 5 in human populations is a major limitation of systemic administration and multiple-dose regimens [98]. Thus, at this time, most OAds can only be administered intratumorally or loco-regional area [10]. To avoid pre-existing immunity, multiple strategies have been pursued, such as using alternative serotypes, exchange of Ad capsid protein, or physically shielding virus particles [99,100,101]. On the other hand, a recent approach employing molecular redirecting of anti-Ad antibodies to tumor cells showed pre-existing antivirus antibodies can also be used as potent anticancer tools [102]. This method comprises the use of a recombinant bifunctional adapter protein with the ability of capturing anti-Ad antibodies but also recognizes tumor cells through a polysialic acid-specific single-chain variable fragment (scFv) [102]. The approach using an retargeted-neutralizing antibody is an attractive option to activate tumors for systemic immunotherapies. Clinical use of immunotherapies as anticancer agents, such as immune checkpoint inhibitors and CAR-T cells, has grown rapidly in the last decade. However, “cold” tumors with few infiltrating T cells are not recognized by the immune system and do not provoke a strong response, resulting in poor overall outcomes and low response rates with immunotherapy. Additionally, the TME serves as a physical/anatomical barrier against immunological therapeutics. Oncolytic virus therapies, including oncolytic adenovirus-based therapy, have long been pursued as a tool for directly killing cancer cells, but recent studies suggest oncolytic viruses can also stimulate local and systemic anticancer immune responses. In addition, viral oncolysis causes destruction of physical/anatomical barriers including the TME and stroma. For these reasons, a combination of oncolytic viruses with immunotherapy is a highly attractive approach. It should be noted that immunostimulatory molecules or immune checkpoint inhibitors used in the context of oncolytic virotherapy will have the added effect of reactivating antiviral immune responses, which normally act to restrict viral replication. It is logical that antiviral immune response induces viral neutralization by humoral immune response and elimination of viral and viral-producing cells by cellular immune response. However, considering the viral proteins are selectively expressed from cancer cells after infection of oncolytic viruses, antiviral cellular immune response is expected to eradicate cancer cells infected with oncolytic viruses. Indeed, the combination of an anti-PD-1 antibody with an intratumorally delivered oncolytic reovirus enhanced the antiviral immunity, but also significantly enhanced the antitumor immune response as well [103]. Antiviral responses might not only inhibit the oncolytic activities of viruses, but at the same time they can activate the antitumor T cell response and prolong the overall survival [104]. Thus, another important challenge is to find the way to manage the perfect balance between antiviral and antitumoral immunity.

The major questions yet to be answered are whether oncolytic viruses can induce strong antitumor immunity through direct oncolysis and tumor antigen release, or if armed viral vectors expressing immuno-enhancers are more efficacious. In addition, immunological, biological, and genetic profiling of cancer patients before, during, and after these therapies will be critical for the advancement of oncolytic virus and immunotherapy combination regimens and their personalization.

## Figures and Tables

**Table 1:** Active clinical trials using adenoviral vectors for cancer immunotherapy.

Ad Vector	Backbone Vector	Transgene	Cancer	Combination Therapy	Clinical Phase	Reference
ETBX-011	Replication-deficient Ad5	CEA	Colon cancer	ETBX-021, ETBX-051, ETBX-061 SBRT, chemotherapy, haNK	I/II	NCT03563157
			Pancreatic cancer	SBRT, chemotherapy, aldoxorubicine HCl, avelumab, bevacizumab, ALT-803 (IL-15), GI-4000,haNK	I/II	NCT03387098
			Advanced cancer	ETBX-051, ETBX-061	I	NCT03384316
Ad5-PSA	Replication-deficient Ad5	PSA	Hormone refractory prostate cancer		II	NCT00583024
			Prostate cancer	ETBX-061, ETBX-051	I	NCT03481816
Ad-E6E7	Replication-deficient Ad5	HPV E6/E7	HPV-associated cancers	MG1-E6E7, atezolizuma	I	NCT03618953
Ad-MAGEA3	Replication-deficient Ad5	MAGE-A3	Advanced/Met. MAGE-A3+ Solid Tumors	MG1MA3 (maraba virus-MAGE-A3)	I/II	NCT02285816
			NSCLC	MG1-MAGEA3, Pembrolizumab	I/II	NCT02879760
Ad5-yCD/mutTKSR39rep-hIL-12	Oncolytic Ad5	Cytosine deaminase, HSV-tK, hIL-12	Prostate cancer		I	NCT02555397
			Metastatic pancreatic cancer	5-FC, standard chemotherapy	I	NCT03281382
Ad-RTS-hIL-12	Replication-deficient Ad5	hIL-12	Pediatric brain tumor	Veledimex	I	NCT03330197
			Glioblastoma, Malignant Glioma	Veledimex	I	NCT02026271
LOAd703	Oncolytic, Ad5/35 fiber	CD40L, 4-1BBL	Pancreatic cancer	Standard chemotherapy	I/II	NCT02705196
			Pancreatic, Biliary, Colorectal, Ovarian	Standard chemotherapy	I/II	NCT03225989
DNX-2440	Oncolytic, *Delta-24, RGD fiber*	OX40L	Glioblastoma		I	NCT03714334
DNX-2401	Oncolytic, *Delta-24, RGD fiber*		Recurrent glioma	Pembrolizumab	II	NCT02798406
ONCOS-102	Oncolytic, *Delta-24,* Ad5/3 fiber	GM-CSF	Melanoma	Cyclophosphamide, Pembrolizumab	I	NCT03003676
TILT-123	Oncolytic, *Delta-24,* Ad5/3 fiber	hTNF-α, hIL-2	Advanced melanoma	Tumor infiltrating lymphocyte (TIL) therapy	I	NCT04217473
